# Visnagin Protects Against Lipopolysaccharide-Induced Acute Kidney Injury by Inhibiting Oxidative Stress and Reducing Ferroptosis

**DOI:** 10.7150/ijms.125642

**Published:** 2026-02-18

**Authors:** Sheng-Wen Wu, Chen-Yu Chiang, Chien-Ying Lee, Shiuan-Shinn Lee, Wen-Ying Chen, Chun-Jung Chen, Ching-Chi Tseng, Yin-Che Lu, Yu-Hsiang Kuan

**Affiliations:** 1 Division of Nephrology, Department of Internal Medicine, Chung Shan Medical University Hospital, Taichung, Taiwan.; 2 Department of Internal Medicine, School of Medicine, Chung Shan Medical University, Taichung, Taiwan.; 3 Department of Pharmacology, School of Medicine, Chung Shan Medical University, Taichung, Taiwan.; 4 Department of Pharmacy, Chung Shan Medical University Hospital, Taichung, Taiwan.; 5 School of Public Health, Chung Shan Medical University, Taichung, Taiwan.; 6 Department of Veterinary Medicine, College of Veterinary Medicine, National Chung Hsing University, Taichung, Taiwan.; 7 Department of Education and Research, Taichung Veterans General Hospital, Taichung, Taiwan.; 8 Department of Dermatology, The Wilshire Lab and Aesthetic Clinic, Shenzhen, China.; 9 Department of Dermatology, Shiso Municipal Hospital, Hyogo, Japan.; 10 Division of Hematology-Oncology, Ditmanson Medical Foundation Chia-Yi Christian Hospital, Chiayi, Taiwan.

**Keywords:** visnagin, lipopolysaccharide, sepsis-associated acute kidney injury (SA-AKI), oxidative stress, ferroptosis

## Abstract

**Background and Objective:**

Sepsis-associated acute kidney injury (SA-AKI) is a life-threatening condition driven by oxidative stress, ferroptosis, and inflammation, yet effective treatments remain unavailable. Visnagin has antioxidant and anti-inflammatory properties and has been traditionally used for cardiovascular and renal disorders, but its role in modulating ferroptosis and redox imbalance in SA-AKI remains unclear. Thus, the study investigated the renoprotective effects of visnagin in a murine lipopolysaccharide (LPS)-induced AKI model, focusing on oxidative stress and ferroptosis.

**Methods:**

A systems pharmacology approach integrating network-based target prediction and molecular docking identified candidate targets. In vivo and in vitro validations in LPS-induced AKI mice and HK-2 cells assessed histopathology, oxidative biomarkers, ferroptosis mediators, and key signaling pathways.

**Results:**

Visnagin demonstrated affinity binding to *AKT1*, *NFE2L2*, *ACSL4*, and *TFRC*, indicating a potential role in modulating oxidative stress and ferroptosis. In vivo, visnagin alleviated renal injury, reduced lipid peroxidation, and downregulated ACSL4 and TfR1 expression, with this accompanied by reduction in renal Fe^2+^ levels. Although visnagin reduced the protein abundance of SOD, catalase, and GSH-Px, it markedly enhanced their enzymatic activities, likely due to decreased oxidative burden and preservation of enzyme functionality under LPS-induced stress. Additionally, visnagin inhibited p-Akt and p-Nrf2, suggesting suppression of upstream signaling pathways. In vitro, visnagin reduced intracellular ROS levels in HK-2 cells and exhibited scavenging activity against various radical species.

**Conclusions:**

Visnagin exerts protective actions through both modulation of Akt/Nrf2 signaling ACSL4/TfR1 signaling and direct free radical scavenging. These findings underscore visnagin's potential as a multitarget therapeutic agent for SA-AKI.

## Introduction

Acute kidney injury (AKI) is among the most prevalent syndromes in hospitalized patients. However, it is often underrecognized despite having substantial implications for clinical outcomes and global health-care burdens 1. Although the Kidney Disease: Improving Global Outcomes (KDIGO) 2012 guidelines define AKI primarily through clinical diagnostic and staging criteria, histopathological assessment remains essential to identifying underlying etiologies and evaluating tubular injury severity 1, 2. Histologically, AKI is characterized by distinct renal alterations, including tubular dilation, intraluminal cast formation, loss of the proximal tubular brush border, vacuolar degeneration of tubular epithelial cells, and glomerular capillary collapse 3, 4. A systematic review of large cohort studies conducted from 2004 to 2012 that encompassed more than 3.5 million individuals reported pooled AKI incidence rates of 21.6% in adults and 33.7% in children, highlighting its widespread prevalence 5, 6. Furthermore, severe AKI (KDIGO stages 2-3) has been consistently associated with increased in-hospital mortality, longer hospital stays, and higher health-care costs 7. In the United States alone, AKI-related admissions contribute an estimated US$5.4 to US$24 billion annually, with these contributing costs largely related to a need for renal replacement therapy, extended intensive care, and frequent readmissions 8. Although advances have been made in supportive care for AKI, few effective therapeutic options are available, indicating an urgent need for novel treatment strategies 9, 10.

Sepsis is the leading cause of AKI in critically ill patients, accounting for approximately 45% to 70% of cases 11-13. Among the pathogenic triggers of sepsis, gram-negative bacterial infections are particularly notable, with lipopolysaccharide (LPS), key components of the outer membrane, serving as a potent initiator of the septic response. LPS-induced AKI is widely used as an experimental model for investigating SA-AKI because it closely replicates the key pathophysiological features observed in human sepsis 14. Oxidative stress plays a central role in LPS-induced AKI; it is characterized by excessive reactive oxygen species (ROS) generation, lipid peroxidation, and reduced activity of antioxidative enzymes, with this ultimately leading to tubular epithelial injury and inflammation 15-17. LPS also upregulates transferrin receptor (TfR1) expression and increases intracellular Fe²⁺ accumulation, thereby promoting ferroptosis, an iron-dependent form of regulated cell death 18, 19. Moreover, LPSs activate Akt phosphorylation, which in turn induces Nrf2 phosphorylation and nuclear translocation, leading to the upregulation of antioxidant enzymes such as HO-1, along with superoxide dismutase (SOD), catalase, and glutathione peroxidase (GSH-Px). This response counteracts ferroptosis and restores redox homeostasis 17, 20.

Visnagin, a natural furanochromone derived from *Ammi visnaga* (L.) Lam., has traditionally been used in various regions to treat conditions such as angina pectoris, myocardial ischemia, hypotension, epileptic seizures, whooping cough, diuresis, and kidney stones 21-23. Its pharmacological activities have since been extended to include anti-inflammatory, antioxidant, anticancer, and antimicrobial effects across multiple disease models 23-27. Notably, visnagin has demonstrated potential as a modulator of key intracellular stress response pathways, particularly the serine/threonine kinase Akt signaling cascade 25. In addition, visnagin has been reported to enhance the Nrf2-mediated antioxidant response and thereby alleviate oxidative stress 28. Excessive reactive oxygen species promote iron-driven lipid peroxidation, whereas Nrf2-governed antioxidant programs can constrain lipid peroxidation and reduce ferroptotic injury. Given its known antioxidant and anti-inflammatory activities, we hypothesized that visnagin would mitigate LPS-induced renal injury by modulating the Akt/Nrf2 axis and suppressing ferroptosis.

## Materials and Methods

### Target Identification of Visnagin Against AKI By Using a Network Pharmacology Approach

AKI-associated genes were curated from CTD, GeneCards, and DisGeNET, whereas putative visnagin targets were predicted using PharmMapper, SwissTargetPrediction, and TargetNet. All identifiers were mapped to UniProt to unify gene/protein annotations. After unifying gene and protein annotations via UniProt, the overlap between AKI-related genes and predicted visnagin targets was defined as the candidate target set for subsequent network analyses, highlighting potential molecular nodes through which visnagin may modulate AKI-related pathology.

### Gene Ontology and Kyoto Encyclopedia of Genes and Genomes Enrichment Analysis

Gene Ontology (GO) enrichment analysis (biological processes, cellular components, and molecular functions) was performed using DAVID, and Kyoto Encyclopedia of Genes and Genomes (KEGG) pathway enrichment was conducted using Enrich. Significantly enriched terms (*P*<.05) were primarily associated with oxidative stress regulation, redox homeostasis, and inflammatory signaling, supporting a mechanistic link between visnagin and AKI-related molecular pathways.

### Protein-Protein Interaction Network Construction

Overlapping targets were imported into the STRING database to construct a protein-protein interaction (PPI) network. The resulting network was visualized and analyzed using Cytoscape software. Key hub genes were identified using topological metrics in the CytoHubba plugin.

### Molecular Docking Analysis

The 3D structures of target proteins, *AKT1*, *ACSL4*, *GSR*, *NFE2L2*, *SOD2*, and *TFRC,* were retrieved from the RCSB Protein Data Bank (PDB). The chemical structures of visnagin and dexamethasone were obtained from the PubChem database. AutoDock 4.0 was used for docking, and the lowest binding energy (kcal/mol) at the annotated binding site was retained for interaction analysis.

### Binding Interaction Analysis

The docking results were visualized using PyMOL and Discovery Studio Visualizer 2025. Hydrogen bonding, hydrophobic interactions, and other noncovalent interactions between ligands and target proteins were analyzed. Binding affinities, expressed in kcal/mol, were recorded to compare interaction strengths. Detailed binding conformations of visnagin with *AKT1*, *ACSL4*, *GSR*, *NFE2L2*, *SOD2*, and *TFRC* were visualized and compared.

### Mouse Model of AKI

A total of 48 male BALB/c mice (4-6 weeks old, 25-30 g) were obtained from the National Center for Biomodels (Taipei, Taiwan). A total of 48 male BALB/c mice (4-6 weeks old, 25-30 g) were used in this study. The sample size was determined based on commonly used designs in LPS-induced AKI models assessing renal histopathology, antioxidant enzyme activities, and protein expression, with two independent experiments conducted using 4 mice per group in each experiment, resulting in n = 8 mice per group for statistical analyses. The animals were housed under controlled conditions, which involved a temperature range of 20°C to 25°C, a 12-h light/dark cycle, and ad libitum access to standard chow and water. All procedures were approved by the Institutional Animal Care and Use Committee (IACUC) of Chung Shan Medical University and adhered to the NIH Guide for the Care and Use of Laboratory Animals (approval number: 2683). Following a 1-week acclimatization period, the mice were randomly assigned to 6 groups (n = 8 per group). The control group received an intraperitoneal (i.p.) injection of dimethyl sulfoxide (DMSO; vehicle for visnagin) 30 min prior to receiving an i.p. injection of sterile phosphate-buffered saline (PBS; vehicle for LPS). The AKI group received DMSO (i.p.) 30 min before receiving a 5 mg/kg i.p. injection of LPS (Cat: L2630, Sigma-Aldrich, MO, USA) to induce AKI. The VIN(5), VIN(10), and VIN(20) groups were pretreated with visnagin (Cat: CFN97314, ChemFace, Hubei, China) at doses of 5, 10, and 20 mg/kg (i.p.), respectively, 30 min prior to administration of LPS. The DEX group received 1 mg/kg dexamethasone (i.p.) 30 min prior to receiving an LPS injection as a positive control. All mice were euthanized at 24 h posttreatment, and their kidneys were harvested for subsequent analysis 29-31.

### Histopathological Analysis of Renal Tubular Damage Through Periodic Acid-Schiff Staining

PAS staining was selected as the primary histological method because it sensitively reflects tubular brush border/glycocalyx integrity and tubular injury severity in LPS-induced AKI. After AKI induction, the mouse kidney tissues were harvested and fixed in 4% paraformaldehyde at 4°C for 24 h to preserve structural integrity. After fixation, the samples were dehydrated through a graded ethanol dehydration series, cleared with xylene, and embedded in paraffin. A rotary microtome was used to cut 5-μm-thick sections and mount them on glass slides. Periodic acid-Schiff (PAS) staining was performed according to standard protocols to visualize the renal tubular structures. PAS-positive areas, which appear purplish-red, were examined under a microscope, and renal fibrosis was evaluated by assessing the distribution and intensity of PAS staining 31, 32. The semiquantitative assessment of kidney injury was quantitatively scored according to a previously reported grading system using a 0-4 scale: 0, no detectable abnormalities; 1, lesions affecting <25% of the examined area; 2, lesions affecting 25-50%; 3, lesions affecting 50-75%; and 4, lesions affecting 75-100% of the area 33.

### Measurement of Antioxidant Enzyme Activities, Lipid Peroxidation, and Ferrous Ion Content

Renal tissues were harvested and rinsed in ice-cold saline, then homogenized in ice-cold PBS (pH 7.4) by using a homogenizer. The homogenates were then centrifuged at 12 000*g* for 20 min at 4°C, and the supernatants were collected for assays. The activities of antioxidant enzymes, including catalase, SOD, and GSH-Px, were quantified using commercially available activity assay kits according to the manufacturers' instructions (catalase, Cat: 707002; SOD, Cat: 706002; GSH-Px, Cat:703102; Cayman, MI, USA). Lipid peroxidation was evaluated by quantifying malondialdehyde (MDA) and 4-hydroxynonenal (4-HNE) by using the TBARS assay kit (Cat: 10009055, Cayman, MI, USA) and 4-HNE ELISA assay kit (Cat: ab238538, Abcam, MA, USA), respectively. Fe²⁺ concentrations were determined using an iron assay kit (Cat: ab83366, Abcam, MA, USA). All assays were performed following the manufacturer's instructions. Protein concentrations in the supernatants were quantified using the Bradford assay, and enzyme activities were normalized to total protein levels 34, 35.

### Western Blotting of Renal Tissues

Renal tissues were homogenized in radioimmunoprecipitation assay buffer supplemented with protease and phosphatase inhibitors. After incubation on ice for 30 min, the lysates were centrifuged at 12 000*g* for 15 min at 4°C to collect the protein-containing supernatant. Protein concentrations were determined using the Bradford assay. Equal amounts of protein were mixed with sodium dodecyl sulfate loading buffer, denatured at 95°C for 5 min, and separated through sodium dodecyl sulfate-polyacrylamide gel electrophoresis. The proteins were subsequently transferred onto polyvinylidene difluoride membranes, which were blocked with 5% nonfat milk for 1 h. The membranes were then incubated with primary antibodies ACSL4 (Cat: SC-365230, Santa Cruz, CA, USA), TfR1 (Cat: SC-393719, Santa Cruz, CA, USA), p-Akt (Cat: SC-514032, Santa Cruz, CA, USA), Akt (Cat: SC-81434, Santa Cruz, CA, USA), p-Nrf2 (Cat: PA5-67520, Invitrogen, Thermo Fisher Scientific, MA, USA), Nrf2 (Cat: SC-365949, Santa Cruz, CA, USA), HO-1 (Cat: SC-390991, Santa Cruz, CA, USA), SOD (Cat: 10269-1-AP, Proteintech, IL, USA), Catalase (Cat: 21260-1-AP, Proteintech, IL, USA), GPX4 (Cat: SC-166570, Santa Cruz, CA, USA) and β-actin (Cat: SC-47778, Santa Cruz, CA, USA) overnight at 4°C. After a wash, the membranes were incubated with horseradish peroxidase-conjugated secondary antibodies for 1 h. After another thorough washing, protein bands were detected using enhanced chemiluminescence reagents and imaged with a FUSION Solo Vision (Vilber, Lourmat, Collégien, France) apparatus. Band intensities were quantified using appropriate analysis software 36, 37.

### Cell Culture and Drug Treatment

HK-2 human renal proximal tubular epithelial cells were cultured in modified Dulbecco's eagle medium supplemented with 10% fetal bovine serum and 1% antibiotic-antimycotic solution. The cells were maintained in a humidified environment containing 95% air and 5% CO_2_ at a controlled temperature of 37°C. For the experiments, the cells were seeded in culture plates and incubated overnight, followed by treatment with visnagin at concentrations of 0, 20, 40, or 80 μM for 1 h. Subsequently, the cells were treated with LPSs at 5 μg/mL or left untreated for 24 h. After treatment, the cells were harvested for analyses of intracellular ROS generation, viability, and antioxidant enzyme activities.

### Cell Viability Assessment

Cell viability was assessed using the MTT reduction assay. Following treatment, the cells were incubated with 5 mg/mL MTT (Cat: AM0815-0001, Bionovas Biotechnology, ON, Canada) for 4 h at 37°C. The medium was then discarded, and dimethyl sulfoxide was added to dissolve the formazan crystals. Subsequently, absorbance was measured at 490 nm by using a microplate reader, with absorbance values reflecting cell viability 35, 38.

### Intracellular ROS Measurement

Following treatment, the cells were incubated with 10 μM DCFH-DA (Cat: 15204, AAT Bioquest, CA, USA) for 30 min at 37°C in the dark. Following this incubation, the cells were washed, and fluorescence intensity was measured using a microplate reader at an excitation/emission wavelength of 488/525nm. ROS levels were presented in terms of the relative fluorescence intensity compared with the control group 38, 39.

### Assessment of Free Radical Scavenging Capacity

The free radical scavenging activity of visnagin was systematically examined using 4 distinct assays. Superoxide radical scavenging was assessed through the NADH-PMS-NBT system, where superoxide anions generated by the NADH (Cat: NB0642, Bio Basic, ON, Canada)-PMS( Cat: P9625, Sigma-Aldrich, MO, USA) reaction reduce NBT (Cat: L11939.06, Thermo Fisher Scientific, MA, USA) to a colored formazan. Visnagin's inhibitory effect on this reaction was quantified by measuring absorbance at 560 nm. The DPPH assay involved incubating visnagin with 0.1 mM DPPH (Cat: D9132, Sigma-Aldrich, MO, USA) methanol for 30 min at room temperature, with this followed by measurement of absorbance decline at 517 nm to determine radical-quenching capacity. In the ABTS assay, ABTS•⁺ radicals were generated by reacting ABTS (Cat: A1888, Sigma-Aldrich, MO, USA) with potassium persulfate (Cat: sc-203362, Santa Cruz, CA, USA) and leaving it to incubate in the dark for 12 h. The resulting radical solution was then diluted to an absorbance of 734 nm before visnagin was added, and the reduction that occurred in absorbance at 734 nm after the reaction indicated ABTS•⁺ scavenging. Hydrogen peroxide scavenging was evaluated by mixing visnagin with 40 mM H₂O₂ in PBS, with the residual H₂O₂ concentration measured through absorbance at 230 nm.

### Statistical Analysis

Data were analyzed using SPSS (IBM, Armonk, NY, USA). Continuous variables are presented as mean ± standard deviation (SD) unless otherwise indicated. All experiments were performed using biological replicates derived from independent experiments (n ≥ 3), and each data point represents an individual biological replicate. Prior to parametric testing, data distributions were assessed for normality using the Shapiro-Wilk test, and homogeneity of variances was evaluated using Levene's test. Group differences were evaluated using one-way analysis of variance (one-way ANOVA), followed by Bonferroni-corrected post hoc tests for multiple comparisons. All statistical tests were 2-tailed, with significance defined as *P* < .05.

## Results

### Network Pharmacology Analysis of the Interaction Between Visnagin and AKI Across 6 Public Databases

**Figure 1:**
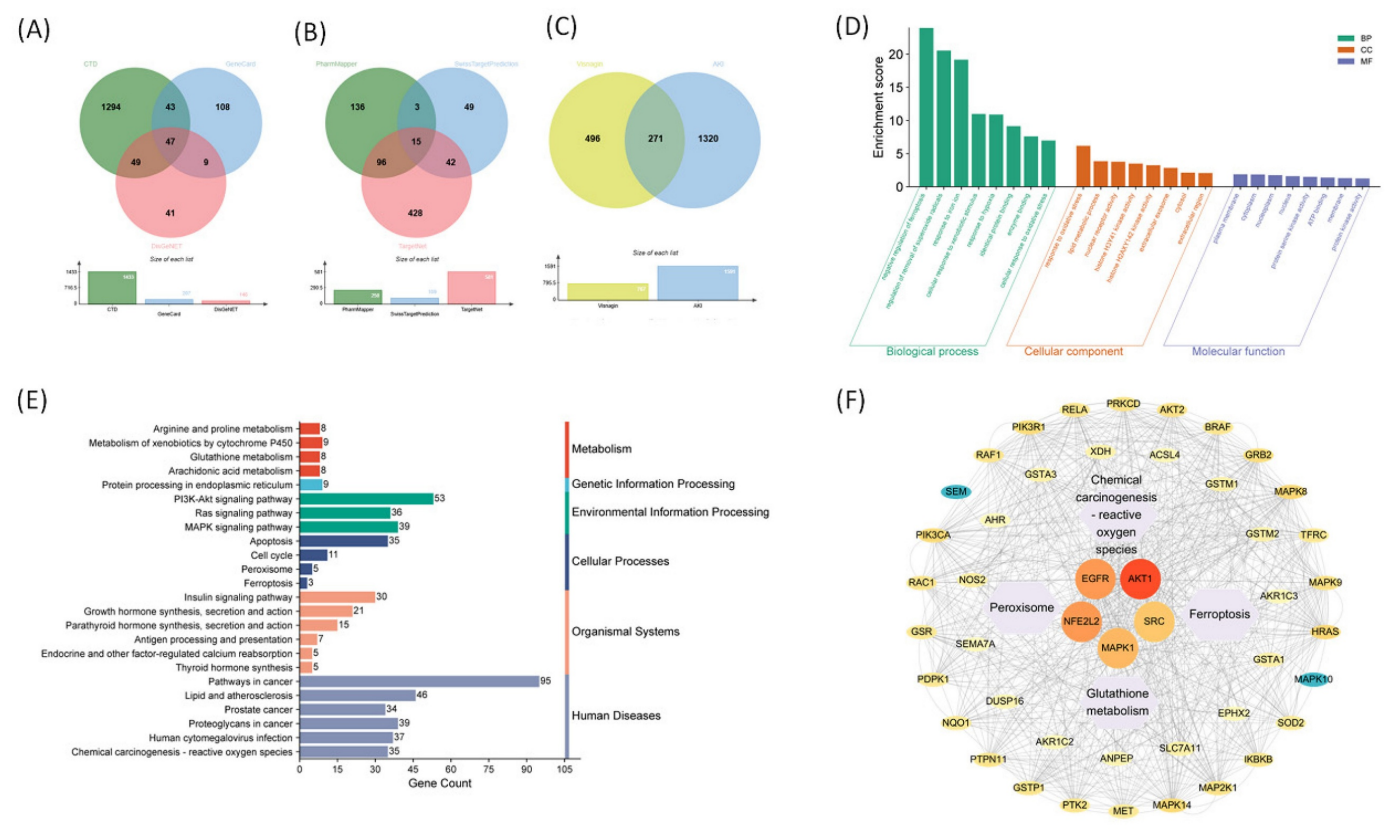
** Network pharmacology analysis of Visnagin targets associated with antioxidant mechanisms and ferroptosis suppression.** (A) Venn diagram of AKI-related genes. (B) Venn diagram of Visnagin targets predicted. (C) Overlapping genes between Visnagin targets and AKI-related genes. (D) GO enrichment analysis targets, categorized by biological process (BP), cellular component (CC), and molecular function (MF). (E) KEGG pathway enrichment analysis. (F) Protein-protein interaction (PPI) network of overlapping genes visualized and key hub genes.

We used a network pharmacology approach to investigate the molecular mechanisms underlying the pharmacological effects of visnagin. A Venn diagram analysis revealed 271 overlapping genes between predicted visnagin targets and AKI-related genes (Figure 1A-C), which were subsequently subjected to a functional enrichment analysis. The GO enrichment analysis (Figure 1D) revealed that these genes were primarily involved in oxidative stress-related biological processes, including responses to ROS, oxidative stress-induced apoptotic signaling, and regulation of cellular redox homeostasis. A cellular component analysis indicated enrichment in membrane-bound organelles, whereas a molecular function analysis highlighted oxidoreductase activity as a key molecular feature. The KEGG pathway enrichment analysis (Figure 1E) further demonstrated that the visnagin-associated targets were significantly enriched in antioxidative and ferroptosis-related signaling pathways, including glutathione metabolism, peroxisome function, PI3K-Akt signaling, and ferroptosis. These results suggest that visnagin may exert cytoprotective effects by modulating oxidative stress responses and inhibiting ferroptosis, thereby offering potential protection against AKI. The PPI network analysis (Figure 1F) revealed key hub targets, notably, including *AKT1* and *NFE2L2*, a regulator of antioxidant defense. Ferroptosis-associated regulators such as *ACSL4* and *TFRC*, along with antioxidative enzymes such as *SOD2* and *GSR*, were localized in the network. These molecules are integral to the regulation of iron metabolism, lipid peroxidation, and ROS detoxification, which underscores the potential role of visnagin in inhibiting ferroptosis and enhancing antioxidant capacity. Collectively, these findings indicate that visnagin confers renal protection by promoting antioxidative defense and suppressing ferroptotic cell death, demonstrating its therapeutic potential in ferroptosis-driven conditions, including AKI.

### Molecular Docking of Visnagin with Antioxidation and Ferroptosis-Related Targets

**Figure 2:**
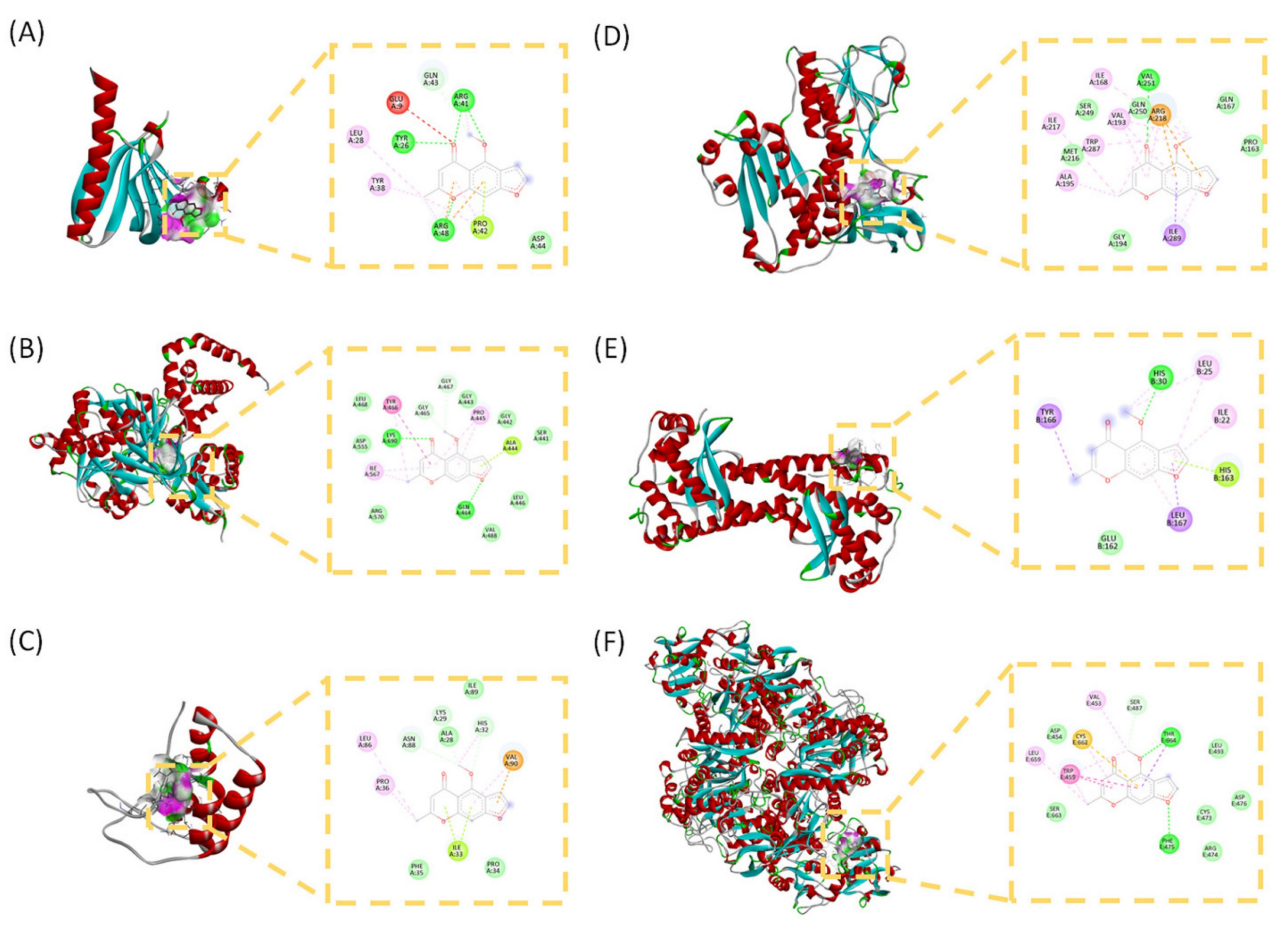
** Binding modes and docking interaction diagrams of Visnagin with *AKT1* (A), *ACSL4* (B), *GSR* (C), *NFE2L2* (D), *SOD2* (E) and *TFRC* (F).** Each panel presents the predicted interaction of Visnagin with the target protein (left: 3D interaction diagram, right: 2D docking pose). Dotted green lines indicate conventional hydrogen bonding interactions, red and yellow dots indicate carbonyl hydrogen bonding interactions, and purple lines denote hydrophobic and π-related interactions.

To investigate the potential molecular interactions of visnagin with targets associated with antioxidation and ferroptosis, molecular docking simulations were conducted on 6 key proteins. Binding affinities were used to evaluate the stability of the ligand-protein interactions. Among the tested targets, visnagin exhibited the strongest binding affinities with *AKT1* (-6.78 kcal/mol; Figure 2A), *ACSL4* (-6.80 kcal/mol; Figure 2B), and *TFRC* (-6.70 kcal/mol; Figure 2C), indicating comparatively favorable docking scores among the tested targets. Conversely, visnagin exhibited moderate to weak binding with *GSR* (-5.85 kcal/mol; Figure 2D), *NFE2L2* (-5.75 kcal/mol; Figure 2E), and *SOD2* (-5.36 kcal/mol; Figure 2F).

### Visnagin Restores Histological Changes in LPS-Induced AKI Mice

**Figure 3:**
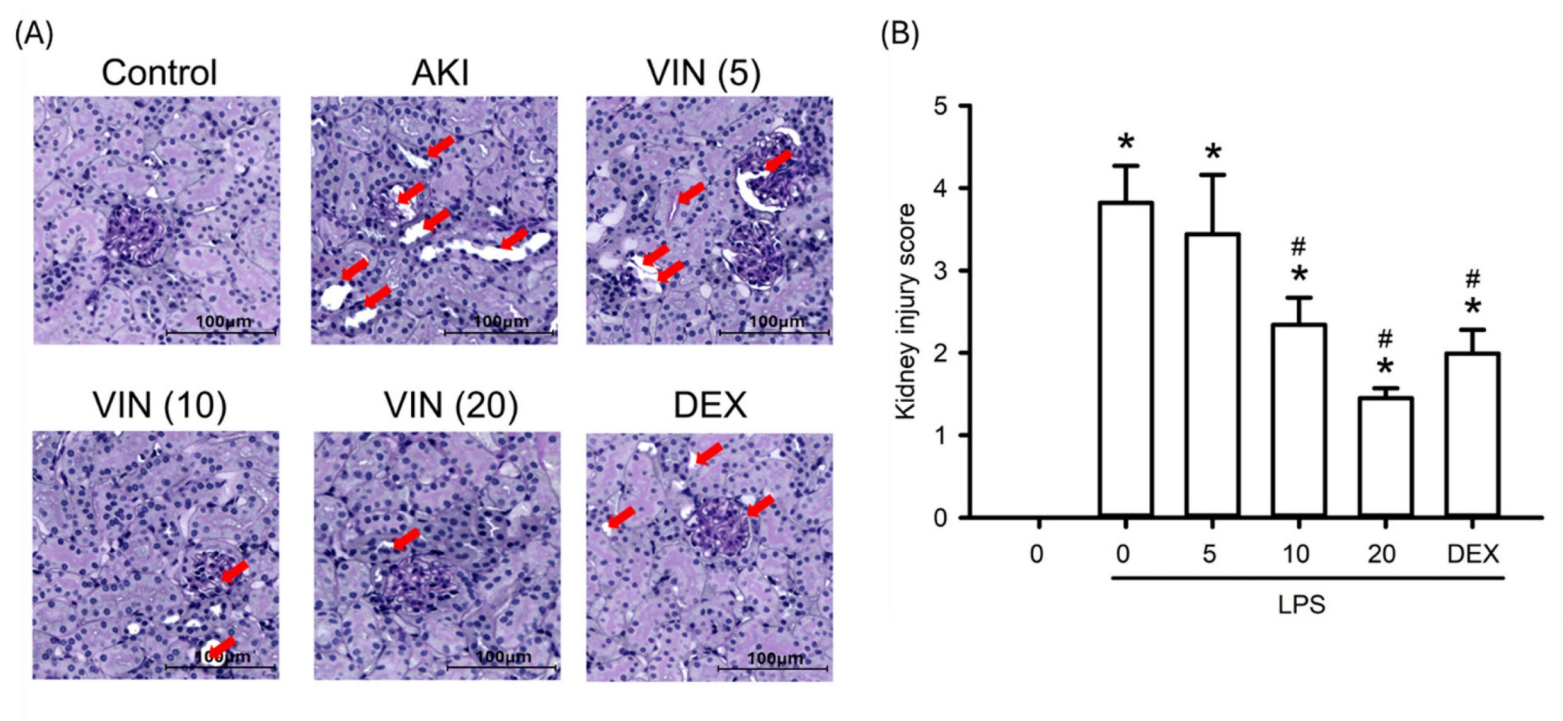
** Visnagin attenuates the histological changes in LPS-induced AKI mice.** Representative PAS-stained kidney sections showing histopathological changes in each experimental group. Tubular injury is indicated by red arrows. Scale bar = 100 μm. Semi-quantitative tubular injury scores were assessed using a 0-4 grading system. Data are presented as mean ± SD from biological replicates derived from independent experiments (n = 3). *P < .05 versus control; #*P* < .05 versus LPS.

To assess the histopathological impact of visnagin on LPS-induced AKI, this study analyzed renal sections from all experimental groups by using PAS staining. LPS administration resulted in marked renal damage, including tubular dilation, intraluminal cast formation, loss of the proximal tubular brush border, glomerular capillary collapse, and vacuolar degeneration of tubular epithelial cells (Figure 3). Treatment with visnagin at 10 and 20 mg/kg markedly ameliorated these pathological changes, as evidenced by improved tubular architecture and preserved tissue integrity. PAS staining further revealed a substantial decrease in the glycocalyx-associated magenta signal along the proximal tubule brush border in LPS-treated mice, which was indicative of brush border disruption; notably, visnagin treatment restored the intensity of this PAS-positive signal, suggesting a protective effect on the tubular surface. Additionally, the results demonstrated a significant increase in the mesangial matrix-to-glomerular area ratio and the mesangial PAS staining density in the LPS group, which indicates mesangial expansion and matrix deposition (Figure 3A). PAS-stained kidney sections were evaluated and semi-quantitatively scored (Figure 3B). LPS challenge induced pronounced histopathological damage, accompanied by a significant increase in the kidney injury score compared with the control group (*P*<.05). Visnagin pretreatment attenuated LPS-induced tubular injury in a dose-dependent manner, with doses of 10 and 20 mg/kg significantly reducing the injury score relative to the LPS group (*P*<.05). These findings indicate that visnagin effectively alleviates LPS-induced renal tubular damage.

### Visnagin Restores Antioxidant Enzyme Activities in Mice With LPS-Induced AKI

**Figure 4:**
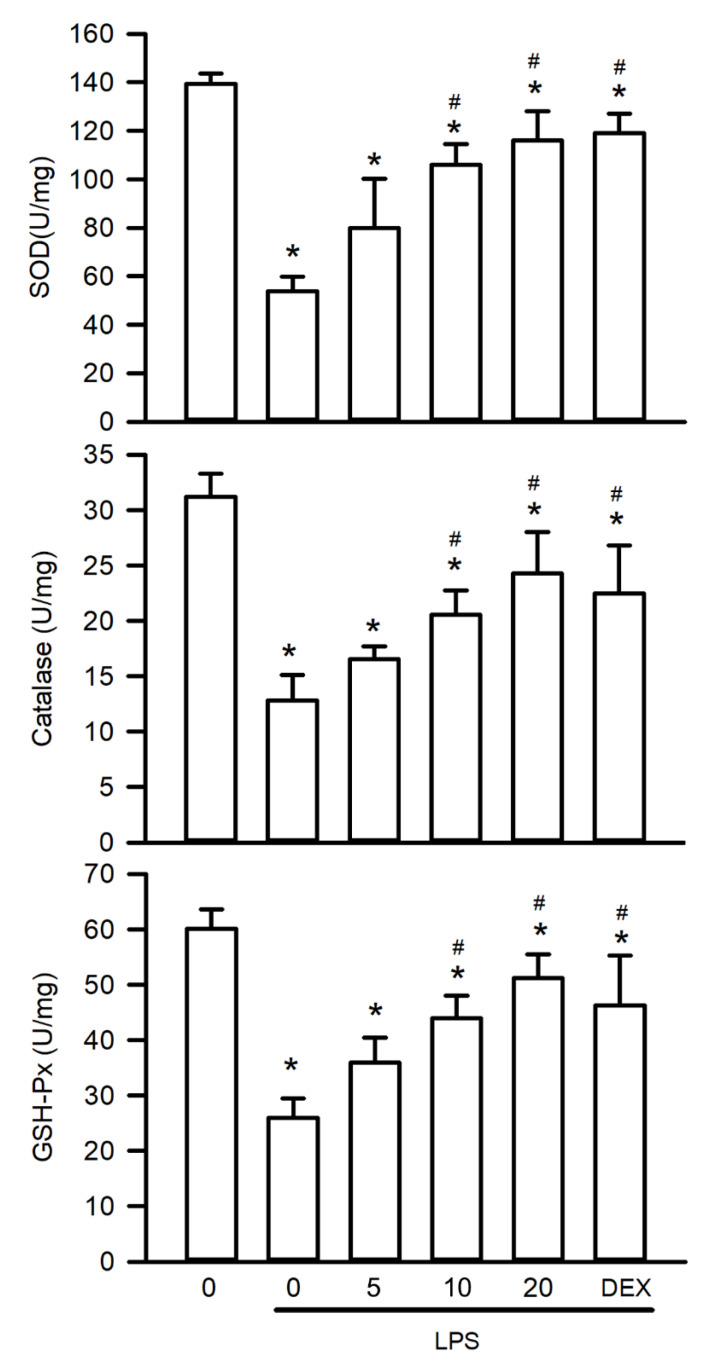
** Visnagin restores antioxidant enzyme activities in LPS-induced AKI.** The activities of SOD, catalase, and GSH-Px were measured by antioxidant enzymes activity assay kits. Results are presented as mean ± SD from biological replicates derived from independent experiments (n = 3). Statistical significance is indicated as *P < .05 versus control; #*P* < .05 versus LPS.

Oxidative stress plays a pivotal role in the pathogenesis of LPS-induced AKI by increasing ROS levels and suppressing activities of antioxidant enzymes. To assess the antioxidative effects of visnagin, this study measured the renal activities of SOD, catalase, and GSH-Px. LPS exposure significantly suppressed the activity of these enzymes, indicating substantial oxidative injury (Figure 4, *P*<.05). Notably, treatment with visnagin at 10 and 20 mg/kg effectively reversed these reductions, restoring antioxidant enzyme function and attenuating oxidative damage in LPS-challenged mice (Figure 4, *P*<.05). This highlights visnagin's protective efficacy with respect to mitigating oxidative stress.

### Visnagin Inhibits Lipid Peroxidation and ACSL4 Expression in Mice With LPS-Induced AKI

**Figure 5:**
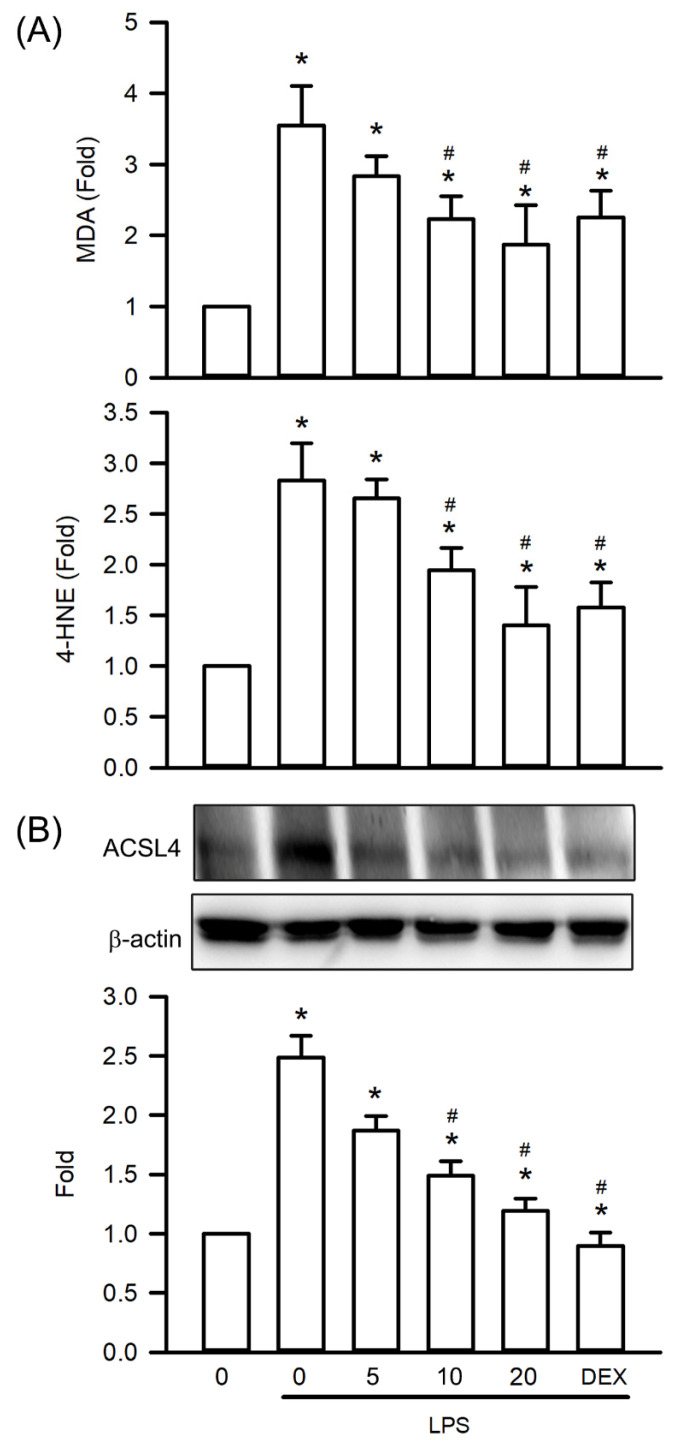
** Visnagin suppresses lipid peroxidation and downregulates ACSL4 expression in renal tissues of LPS-induced AKI mice.** Lipid peroxidation levels were assessed by measuring MDA and 4-HNE concentrations using commercially available assay kits. In addition, the expression of ACSL4 was measured by western blot assay. Results are presented as mean ± SD from biological replicates derived from independent experiments (n = 3). Statistical significance is indicated as *P < .05 versus control; #*P* < .05 versus LPS.

To investigate the protective effects of visnagin against oxidative stress in LPS-induced AKI, this study assessed lipid peroxidation markers and ACSL4 expression in renal tissues obtained from mice with LPS-induced AKI. LPS treatment significantly increased MDA and 4-HNE levels, indicating increased lipid peroxidation compared with that in controls (Figure 5A, *P*<.05). Visnagin administration at 10 and 20 mg/kg significantly counteracted these increases, indicating an ability to mitigate oxidative damage to membrane lipids (Figure 5A, *P*<.05). Additionally, a western blot analysis revealed a marked upregulation of ACSL4 expression in the kidneys of LPS-treated mice. Visnagin pretreatment at the aforementioned doses significantly suppressed ACSL4 expression relative to that in the LPS group (Figure 5B, *P*<.05). These findings suggest that visnagin not only mitigates lipid peroxidation but also inhibits ACSL4-mediated remodeling of membrane phospholipids in the context of LPS-induced AKI (Figure 5B, *P*<.05).

### Visnagin Inhibits Fe^2+^ Accumulation and TfR1 Expression in Mice With LPS-Induced AKI

**Figure 6:**
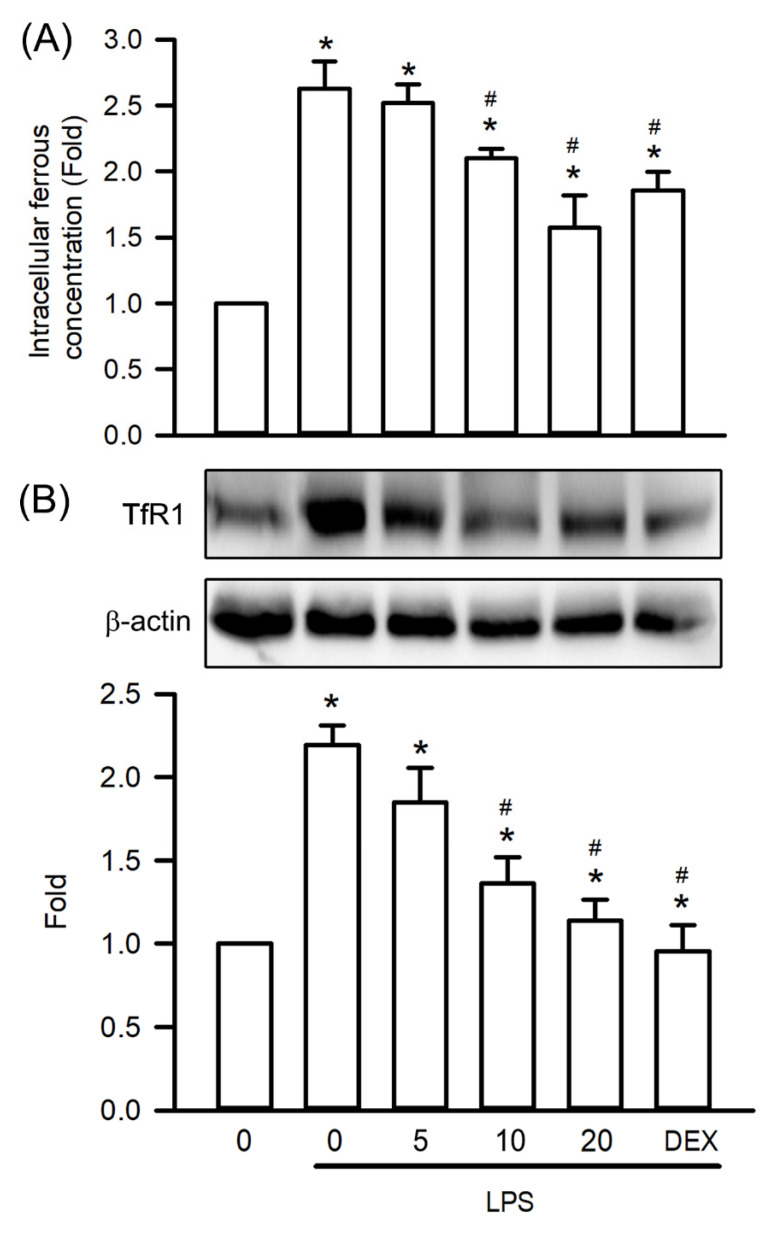
** Visnagin suppresses Fe^2+^ accumulation and downregulates TfR1 expression in renal tissues of LPS-induced AKI mice.** The level of Fe^2+^ accumulation was measured by the iron assay kit. In addition, the expression of TfR1 was measured by western blot assay. Data are presented as mean ± SD from biological replicates derived from independent experiments (n = 3). Statistical significance is indicated as *P < .05 versus control; #*P* < .05 versus LPS.

To investigate visnagin's protective effects against ferroptosis, a form of regulated cell death driven by oxidative stress, in LPS-induced AKI, this study assessed renal Fe²⁺ levels and TfR1 expression in mice with LPS-induced AKI. LPS administration significantly increased Fe²⁺ accumulation in the kidney tissues compared with that in controls, indicating elevated intracellular iron availability that promotes lipid peroxidation (Figure 6A, *P*<.05). Notably, visnagin treatment at 10 and 20 mg/kg significantly attenuated Fe²⁺ accumulation, suggesting a capacity to alleviate iron overload (Figure 6A, *P*<.05). Furthermore, a western blot analysis revealed a substantial upregulation of TfR1 expression following LPS administration, which is consistent with the finding of increased iron uptake (Figure 6B, *P*<.05). Notably, visnagin pretreatment at both doses effectively suppressed TfR1 expression (Figure 6B, *P*<.05). These findings indicate that visnagin at least partly confers renoprotection in LPS-induced AKI by reducing Fe²⁺ accumulation and limiting TfR1-mediated iron transport, thereby mitigating ferroptotic stress.

### Visnagin Inhibits Akt and Nrf2 Phosphorylation and Antioxidative Enzyme Expression in Mice With LPS-Induced AKI

**Figure 7:**
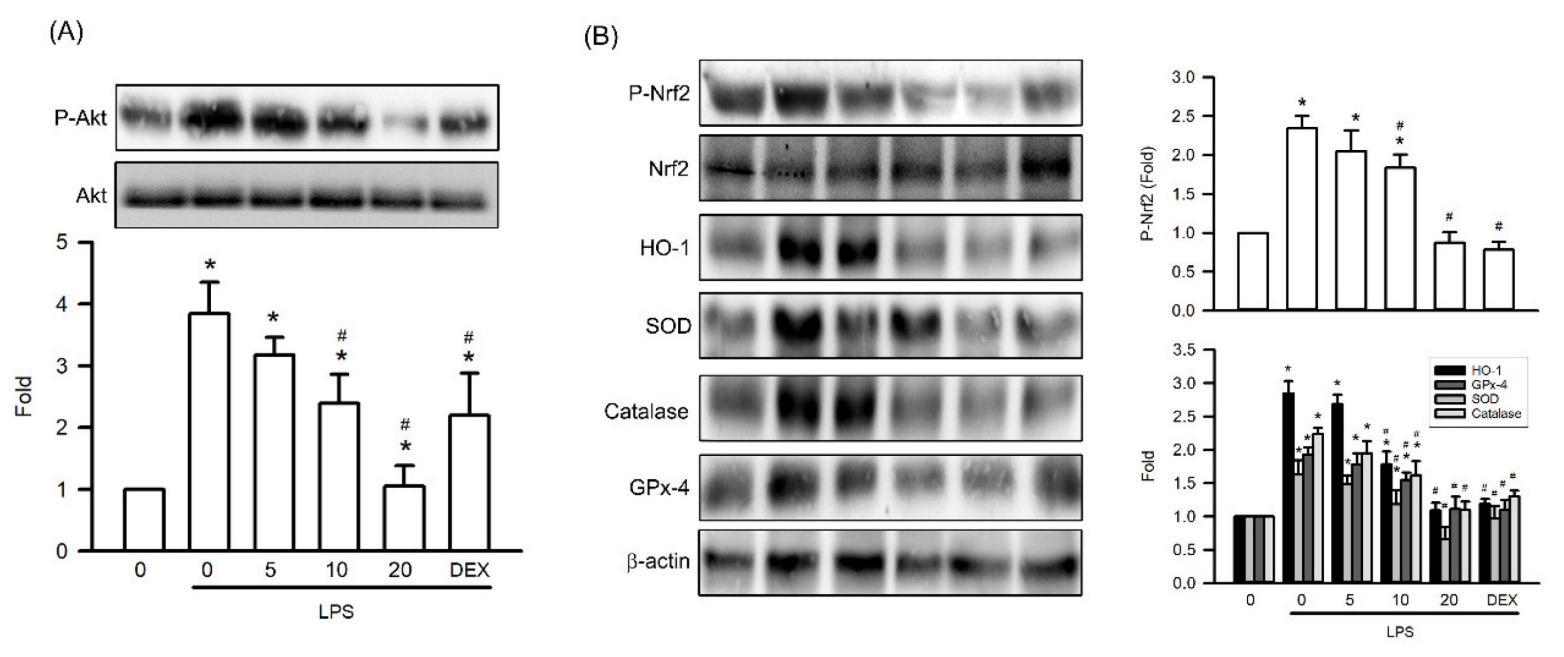
** Visnagin suppresses Akt and Nrf2 phosphorylation and downregulates antioxidative enzyme expression in renal tissues of LPS-induced AKI mice.** Representative western blot images and quantitative analysis of phosphorylated Akt, phosphorylated Nrf2, and antioxidant-related proteins (HO-1, SOD, catalase, and GPX4) in renal tissues. Protein expression levels were normalized to the corresponding loading controls. Data are presented as mean ± SD from biological replicates derived from independent experiments (n = 3). Statistical significance is indicated as *P <.05 versus control; #*P* <.05 versus LPS.

To elucidate the molecular mechanisms underlying visnagin's protective effects in LPS-induced AKI, we assessed the renal phosphorylation levels of Akt and Nrf2, along with the expression of key antioxidant enzymes. LPS exposure significantly increased Akt phosphorylation compared with that in controls, indicating activation of prosurvival signaling under inflammatory conditions (Figure 7A, *P*<.05). Visnagin pretreatment at 10 and 20 mg/kg markedly suppressed Akt phosphorylation, indicating modulation of this pathway in response to LPS (Figure 7A, *P*<.05). Furthermore, because Nrf2 operates downstream of Akt and regulates the expression of antioxidant enzymes such as HO-1, SOD, catalase, and GSH-Px, we also assessed these targets. Consistent with the findings regarding Akt activation, LPS induced substantial upregulation of Nrf2 phosphorylation and antioxidant enzyme expression, whereas visnagin treatment markedly attenuated these responses (Figure 7B, *P*<.05). These findings suggest that the protective effects of visnagin involve suppression of the Nrf2 antioxidative axis through Akt phosphorylation.

### Visnagin Exhibits Free Radical Scavenger Activity In Vivo and In Vitro

**Figure 8:**
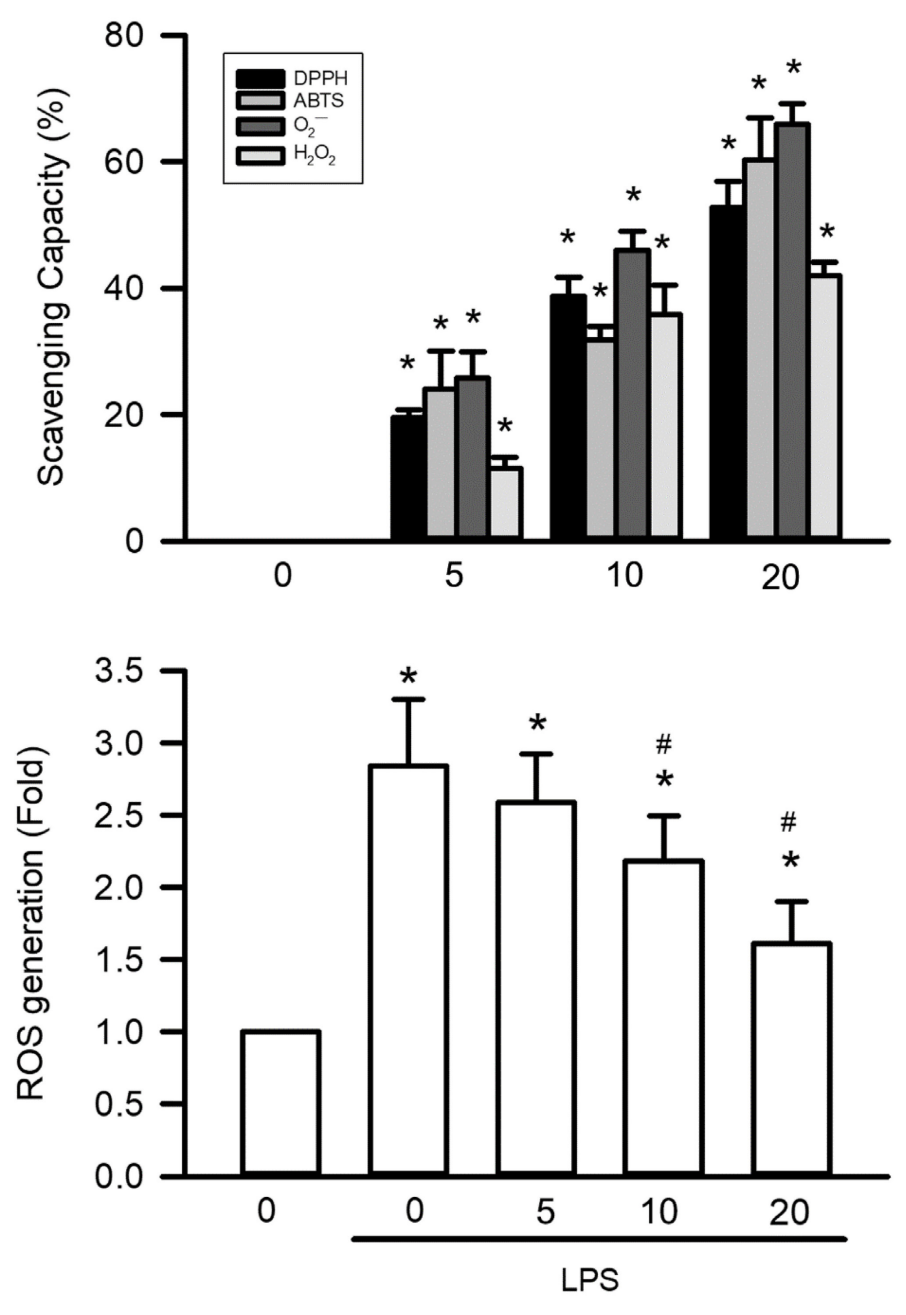
** Visnagin exhibits free radical scavenger activity in vivo and in vitro.** To evaluate the antioxidant potential of visnagin, a comprehensive panel of in vitro free radical scavenging assays was performed, including NADH-PMS-NBT assay, DPPH assay, ABTS assay, and H₂O₂ scavenging assay. For in vivo validation, the level of intracellular ROS was assessed using DCFH-DA fluorescence targeting in HK2 cells. Results are presented as mean ± SD from biological replicates derived from independent experiments (n = 5). Statistical significance is indicated as *P < .05 versus control and #*P* < .05 versus LPS.

The antioxidant capacity of visnagin was systematically assessed using 4 in vitro assays targeting different reactive species. At a concentration of 20 μM, visnagin significantly reduced superoxide anions in the NADH-PMS-NBT assay, nitrogen-centered radicals in the DPPH assay, ABTS•⁺ cations in the ABTS assay, and hydrogen peroxide in the H₂O₂ scavenging assay (Figure 8A, *P*<.05). These effects were concentration-dependent, indicating visnagin has broad-spectrum antioxidant activity, even at low micromolar levels, and a capacity to neutralize multiple reactive species. To validate these findings in a cellular context, HK-2 renal tubular epithelial cells were treated with LPSs to induce oxidative stress, and intracellular ROS levels were assessed using DCFH-DA fluorescence. LPS exposure markedly increased ROS accumulation, whereas visnagin pretreatment at 20 μM significantly reduced DCF fluorescence intensity in a dose-dependent manner, indicating effective suppression of intracellular oxidative stress (Figure 8B, *P*<.05). Together, these results confirm that visnagin exhibits potent free radical scavenging activity both in vitro and in cellular models, with these findings supporting its role in mitigating LPS-induced oxidative damage.

## Discussion

AKI is a critical global health concern, with an estimated 13 million new cases and approximately 1.7 million deaths related to the condition occurring annually; it is associated with a substantial clinical and socioeconomic burden. In this study, we employed an integrated network pharmacology and molecular docking strategy to elucidate the protective mechanisms of visnagin against LPS-induced AKI, focusing on its antioxidative and antiferroptotic effects 1, 40, 41. The network pharmacology analysis revealed 271 overlapping genes between the predicted visnagin targets and AKI-related genes, indicating a substantial pharmacological overlap. The GO enrichment analysis elucidated key biological processes, including response to ROS, oxidative stress-induced apoptotic signaling, and regulation of redox homeostasis. The molecular function analysis revealed oxidoreductase activity, whereas the KEGG pathway analysis implicated glutathione metabolism, peroxisome signaling, PI3K-Akt signaling, and ferroptosis as central to visnagin's mechanisms of action. Furthermore, PPI network analysis revealed key hub targets, including *AKT1*, *NFE2L2*, *ACSL4*, *TFRC*, *SOD2*, and *GSR*—molecules closely linked to iron metabolism, lipid peroxidation, and antioxidant defense. Molecular docking simulations further supported these findings, demonstrating strong binding affinities between visnagin and *AKT1* (-6.78 kcal/mol), *ACSL4* (-6.80 kcal/mol), and *TFRC* (-6.70 kcal/mol), indicating potential for stable ligand-protein complex formation. To contextualize the modest docking scores (~-6 kcal/mol), we compared them with experimentally characterized binding affinities or biochemical potencies of representative ligands and inhibitors relevant to our predicted targets. Holo-transferrin binds TfR1 with very high affinity, reflecting that canonical physiological receptor-ligand interactions can reach sub-nanomolar to low-nanomolar ranges 42. Similarly, in the Nrf2 axis, the Keap1 Kelch domain binds the Nrf2 ETGE-containing peptide with low-nanomolar affinity 43. In contrast, small-molecule enzyme inhibitors, such as reported ACSL4 inhibitors, typically show biochemical potencies in the submicromolar range 44, while allosteric Akt inhibitors such as MK-2206 exhibit nanomolar potency against Akt1 45. Taken together, the docking scores observed in this study are consistent with a hit-like to moderate binding regime, supporting plausible target engagement and binding poses rather than implying high-affinity interactions based solely on docking results. In *AKT1*, visnagin formed hydrogen bonds with GLU9, ARG41, ARG48, and GLN43, along with hydrophobic and π-π stacking interactions involving TYR26, PRO42, and TYR38, which closely resembles the binding mode of known Akt inhibitors. These interactions suggest that visnagin may modulate Akt phosphorylation and subsequent Nrf2 activation, which is a pivotal axis in cellular antioxidant defense. For *ACSL4*, visnagin occupied the acyl-CoA substrate-binding cleft, engaging in hydrogen bonding with GLN464, ALA444, and GLY445 and forming π-π and electrostatic interactions with TYR466, LYS690, and ILE567, which indicates its potential to inhibit ACSL4 activity and suppress lipid peroxidation and ferroptotic cell death. Visnagin also bound to *TFRC* through hydrogen bonds with THR664 and PHE475, with this occurring along with π-π stacking and hydrophobic interactions with TRP459 and CYS662, suggesting interference with transferrin binding and regulation of iron homeostasis. These findings collectively support visnagin's ability to modulate Akt signaling, lipid peroxidation, and iron metabolism through direct molecular interactions, thereby contributing to its multifaceted renoprotective effects in LPS-induced AKI.

SA-AKI is increasingly being recognized as resulting from converging pathophysiological processes, including systemic inflammation, oxidative stress, endothelial dysfunction, and ferroptosis 10, 46-48. Consistent with this understanding, our LPS-induced AKI model demonstrated extensive renal structural damage, characterized by tubular epithelial injury, brush border loss, tubular lumen dilation, intraluminal cast formation, and glomerular capillary rarefaction. These histopathological changes were confirmed through PAS staining, which revealed marked depletion of the glycocalyx-rich brush border, a hallmark of severe tubular injury 3, 4, 46. Moreover, pretreatment with visnagin markedly attenuated these structural abnormalities, demonstrating visnagin's protective effect against LPS-induced renal damage. These findings are consistent with those of studies demonstrating that visnagin mitigates histopathological damage in glycerol-induced AKI, a model of rhabdomyolysis-associated kidney injury 49. That study also reported that visnagin is able to restore the activity of antioxidant defense enzymes, such as SOD, catalase, and GSH-Px, which are otherwise suppressed by glycerol-induced oxidative injury 49. Similarly, visnagin has been reported to alleviate cognitive dysfunction in ischemia-reperfusion cerebral injury and myocardial damage in isoproterenol-treated rats by restoring antioxidant enzyme activities in the brain 50, 51. In our study, LPS administration significantly reduced the activities of antioxidant defense enzymes, which was reversed by visnagin in the kidneys of AKI mice. These results suggest that oxidative stress plays a crucial role in the pathogenesis of LPS-induced AKI and that visnagin exerts renoprotective effects by enhancing the activity of key antioxidant defense enzymes.

In SA-AKI, excessive ROS production disrupts redox homeostasis, leading to pronounced lipid peroxidation in renal tubular cells. MDA and 4-HNE, key byproducts of this process, are widely recognized as being reliable biomarkers of oxidative damage 51-53. MDA, a mutagenic aldehyde formed during the degradation of polyunsaturated fatty acids, reflects cumulative oxidative stress, whereas 4-HNE not only acts as a cytotoxic product of lipid peroxidation but also promotes mitochondrial dysfunction and apoptotic cell death. This aldehyde-mediated damage exacerbates tubular injury in SA-AKI 54-56. ACSL4 further enhances the susceptibility of renal tubular cells to ferroptosis by catalyzing the incorporation of arachidonic acid into phospholipids, which are prone to ROS-induced peroxidation. This ACSL4-mediated lipid remodeling intensifies oxidative damage and contributes to ferroptotic cell death in SA-AKI 57, 58. Research has demonstrated that visnagin reduces MDA formation in models of glycerol-induced AKI, ischemia-reperfusion cerebral injury, and isoproterenol-induced myocardial injury 50, 51. Consistently, our study demonstrated that visnagin significantly reduced MDA accumulation in LPS-induced AKI mice. Notably, this study is the first to report that visnagin also suppresses 4-HNE production and downregulates ACSL4 expression. These findings support the hypothesis that visnagin exerts renoprotective effects in SA-AKI by attenuating lipid peroxidation and inhibiting ACSL4-mediated ferroptosis.

TfR1 is a key regulator of cellular iron uptake and has been increasingly recognized as a pivotal mediator of ferroptosis 59, 60. By facilitating the internalization of transferrin-bound Fe³⁺, TfR1 promotes intracellular iron accumulation. Once internalized, Fe³⁺ is rapidly reduced to Fe²⁺, which expands the labile iron pool LIP 60, 61. Elevated Fe²⁺ levels catalyze Fenton reactions, generating hydroxyl radicals that drive lipid peroxidation and ferroptotic cell death 60, 61. In LPS-induced AKI, TfR1 upregulation is accompanied by increased renal Fe²⁺ concentrations, which exacerbates oxidative stress and tubular damage 62, 63. In this study, LPS-induced AKI resulted in marked increases in both TfR1 expression and renal Fe²⁺ levels, which is consistent with enhanced iron accumulation and ferroptosis activation. Notably, visnagin significantly suppressed TfR1 expression and Fe²⁺ concentrations, indicating that its renoprotective effects may involve suppression of iron overload. By limiting Fe²⁺ availability, visnagin may inhibit Fenton chemistry-mediated ROS production and subsequent lipid peroxidation, thereby disrupting the key mechanism of ferroptosis. These findings provide novel mechanistic insights into visnagin's renoprotective role, highlighting its potential to inhibit ferroptosis through the modulation of iron metabolism and oxidative stress pathways in LPS-induced AKI.

Following LPS or CLP treatment, Akt was significantly phosphorylated in the renal tissues of mice with AKI 64-66. Akt subsequently phosphorylated downstream effectors such as Nrf2, which drives the expression of antioxidant enzymes such as HO-1, SOD, catalase, and GSH-Px 67, 68. Consistent with other studies, our study confirmed that LPS administration induces pronounced activation of Akt in the kidney 65, 69, as evidenced by the increased phosphorylation of both Akt and Nrf2, along with upregulation of HO-1, SOD, catalase, and GPX4. Although this cascade is traditionally associated with adaptative responses to oxidative stress, emerging evidence indicates that in the context of sepsis or systemic inflammation, sustained or dysregulated activation of the Akt/Nrf2 axis may promote maladaptive outcomes, including proinflammatory signaling, oxidative damage, and iron-dependent toxicity 70, 71. Consistent with these notions, our findings revealed that visnagin treatment markedly suppressed LPS-induced phosphorylation of Akt and Nrf2 as well as HO-1 expression. This suppression corresponded to reduced levels of oxidative damage markers, such as MDA and 4-HNE, and downregulation of ferroptosis-associated proteins, including ACSL4 and TfR1. Notably, HO-1 catalyzes the degradation of heme into biliverdin, carbon monoxide, and Fe²⁺, with the latter being a critical driver of lipid peroxidation and ferroptosis 72, 73. Therefore, visnagin-mediated inhibition of HO-1 may help prevent iron overload and subsequent ROS generation through Fenton chemistry, thereby alleviating tubular injury. Furthermore, the observed reduction in Nrf2 phosphorylation by visnagin suggests that excessive or prolonged Nrf2 activation during endotoxemia may shift from a protective role to a pathological one, potentially by disrupting redox homeostasis and cellular iron metabolism.

LPS exposure in sepsis triggers a burst of ROS production in renal tissues. This oxidative surge arises from enzymatic sources activated during endotoxemia 74. In turn, elevated ROS levels initiate redox signaling that activates the PI3K/Akt pathway 75. Thus, LPS sets off an ROS-Akt feed-forward loop that endotoxin-driven ROS not only inflict direct cellular damage but also serve as second messengers that activate Akt kinase signaling. Once activated by ROS, phospho-Akt propagates survival signaling that includes upregulation of the antioxidant response. A key downstream target is the transcription factor Nrf2. Activated Akt has been shown to promote Nrf2 activation by phosphorylating and inhibiting GSK-3β, a kinase that otherwise targets Nrf2 for degradation 76. With GSK-3β restrained, Nrf2 evades cytosolic sequestration and translocates into the nucleus. In essence, p-Akt serves as a molecular switch that releases Nrf2 from its inhibitor, enabling Nrf2 phosphorylation and nuclear accumulation. Once in the nucleus, p-Nrf2 binds antioxidant response elements and drives transcription of a battery of cytoprotective genes 77. This Akt-Nrf2 crosstalk represents a critical adaptive mechanism in SA-AKI as ROS levels rise, Akt activation pushes Nrf2 to bolster the cell's antioxidant defenses, ostensibly to restore redox homeostasis.

A particularly notable finding of this study is that visnagin treatment significantly enhanced the enzymatic activities of key antioxidant enzymes—SOD, catalase, and GSH-Px—despite concurrently reducing their protein expression levels. This apparent disconnect between protein abundance and catalytic function suggests that visnagin restores redox homeostasis not by upregulating antioxidant gene expression but by preserving or enhancing the enzymatic activity of existing antioxidant enzymes. Importantly, enzymatic activity is highly susceptible to oxidative inactivation; therefore, lowering the oxidative burden can restore catalytic function even without increasing protein abundance. Such effects may be mediated through posttranslational mechanisms or conformational stabilization under oxidative stress. This paradox can be further explained by the direct free radical scavenging capacity of visnagin, as confirmed by its ability to neutralize ABTS, DPPH, superoxide, and hydrogen peroxide in cell-free systems. Moreover, visnagin markedly reduced intracellular ROS accumulation in LPS-treated HK-2 cells. These findings suggest that visnagin reduces the oxidative burden, thereby preventing the inactivation of antioxidant enzymes and enabling the recovery of their catalytic efficiency, even in the context of reduced protein expression. Thus, the increase in SOD, catalase, and GSH-Px activities is consistent with function-preserving mechanism rather than canonical Nrf2-driven transcriptional induction.

**Figure 9:**
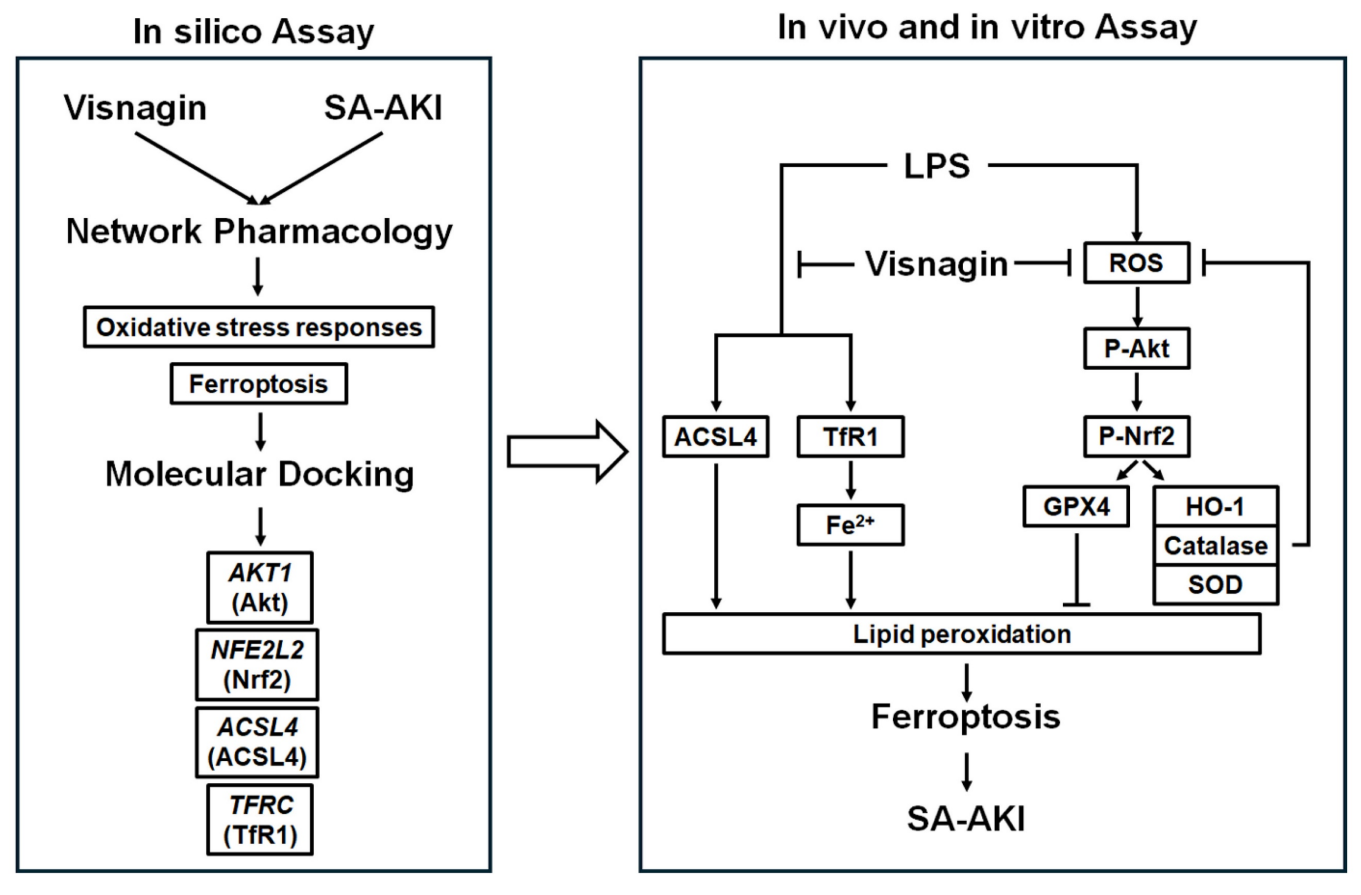
** Mechanisms of visnagin against LPS-induced SA-AKI.** Network pharmacology and molecular docking identified *AKT1*, *NFE2L2* (Nrf2), *ACSL4*, and *TFRC* (TfR1) as key targets. In the proposed model, LPS activates Akt/Nrf2 signaling, increases ROS, and promotes TfR1/Fe²⁺ and ACSL4 driven lipid peroxidation, leading to ferroptosis and SA-AKI. Visnagin suppresses Akt/Nrf2 phosphorylation, reduces ROS, downregulates ACSL4 and TfR1, and limits lipid peroxidation, thereby alleviating ferroptosis and renal injury.

This dissociation between enzyme expression and activity suggests a functional enhancement of the antioxidant defense system, which is likely achieved through the preservation of enzyme structure against oxidative inactivation or the stabilization of favorable posttranslational redox states. Therefore, the increased activities of SOD, catalase, and GPX may represent a secondary effect of visnagin's ROS-scavenging capacity, which mitigates enzyme damage and supports cellular redox homeostasis under septic stress. Importantly, this study provides the first mechanistic evidence that visnagin directly modulates ferroptosis through coordinated regulation of the Akt/Nrf2 axis and iron-lipid peroxidation pathways in LPS-induced AKI. This interpretation also helps reconcile our signaling results. In endotoxemia, Nrf2 signaling activation can be protective, whereas sustained or dysregulated activation may become maladaptive and fail. Nrf2 downstream branches can promote iron liberation through heme degradation, increasing labile Fe²⁺ and thereby amplifying Fenton chemistry, lipid peroxidation, and ferroptotic stress 78. Accordingly, the visnagin-associated normalization of Akt and Nrf2 phosphorylation likely represents a recalibration of maladaptive endotoxemic redox signaling rather than a blockade of a protective axis. Whereas previous studies largely characterized visnagin as a general effect of antioxidative or antiinflammation 79, 80, our data mechanistically connect visnagin to ferroptosis suppression and Akt/Nrf2 pathway tuning in the context of LPS-induced AKI. Importantly, our findings extend the established antioxidant profile of visnagin by linking it to ferroptosis control and Akt/Nrf2 pathway tuning under LPS challenge, suggesting that visnagin acts at dual complementary levels by direct radical-scavenging activity and preservation of endogenous antioxidant capacity. Together, this dual-layer redox regulation provides a mechanistic basis for the potent renoprotective efficacy of visnagin in LPS-induced AKI and highlights its novelty beyond a conventional antioxidant description (Figure 9).

## Conclusions

This study provides preliminary evidence that visnagin exerts renoprotective effects on a mouse model of LPS-induced AKI by modulating pathways involved in oxidative stress and ferroptosis. Network pharmacology and molecular docking analyses revealed AKT, Nrf2, ACSL4, and TfR1 as key targets of visnagin. In vivo, visnagin attenuated renal histological damage; inhibited Akt and Nrf2 phosphorylation; and reduced the expression of HO-1, SOD, catalase, and GPX4. Notably, despite a reduction in protein levels, the enzymatic activities of these antioxidants were restored. This discrepancy was corroborated by in vitro and intracellular assays, in which visnagin effectively scavenged ROS in chemical systems and reduced intracellular oxidative stress, which suggests that its antioxidant action preserves enzyme function through nongenomic mechanisms. Additionally, visnagin downregulated ACSL4 and TfR1 expression and reduced Fe²⁺ accumulation and lipid peroxidation, indicating it plays a role in mitigating ferroptosis. Collectively, these results suggest that visnagin modulates multiple levels of oxidative and iron-related injury pathways.

## Data Availability

The datasets generated and analyzed during the current study are available from the corresponding author upon reasonable request. The molecular docking data, network pharmacology results, and raw experimental data from in vivo and in vitro assays are archived in institutional repositories and can be provided to qualified researchers for verification and further analysis.
